# Highly Omnidirectional and Frequency Controllable Carbon/Polyaniline-based 2D and 3D Monopole Antenna

**DOI:** 10.1038/srep13615

**Published:** 2015-09-04

**Authors:** Keun-Young Shin, Minkyu Kim, James S. Lee, Jyongsik Jang

**Affiliations:** 1 World Class University program of Chemical Convergence for Energy & Environment, School of Chemical and Biological Engineering, Seoul National University, 151-742, Korea; 2 A Photo-Electronic Hybrids Research Center, Korea Institute of Science and Technology (KIST), Seoul 136-791, Korea

## Abstract

Highly omnidirectional and frequency controllable carbon/polyaniline (C/PANI)-based, two- (2D) and three-dimensional (3D) monopole antennas were fabricated using screen-printing and a one-step, dimensionally confined hydrothermal strategy, respectively. Solvated C/PANI was synthesized by low-temperature interfacial polymerization, during which strong π–π interactions between graphene and the quinoid rings of PANI resulted in an expanded PANI conformation with enhanced crystallinity and improved mechanical and electrical properties. Compared to antennas composed of pristine carbon or PANI-based 2D monopole structures, 2D monopole antennas composed of this enhanced hybrid material were highly efficient and amenable to high-frequency, omnidirectional electromagnetic waves. The mean frequency of C/PANI fiber-based 3D monopole antennas could be controlled by simply cutting and stretching the antenna. These antennas attained high peak gain (3.60 dBi), high directivity (3.91 dBi) and radiation efficiency (92.12%) relative to 2D monopole antenna. These improvements were attributed the high packing density and aspect ratios of C/PANI fibers and the removal of the flexible substrate. This approach offers a valuable and promising tool for producing highly omnidirectional and frequency-controllable, carbon-based monopole antennas for use in wireless networking communications on industrial, scientific, and medical (ISM) bands.

Rapid advances in wireless communications using radio frequency (RF) antennas to transfer data have led to the development of ubiquitous sensor networks that build on the embedding of small devices into daily objects as means of ensuring security, environmental monitoring, and increasing personal productivity^1^,^2^. Therefore, large efforts have been devoted to the fabrication of flexible, miniaturized, and reliable antennas^3^. The implementation of high efficiency, omnidirectional antennas is a particularly critical issue for wireless networks due to their relatively low cost, simple processing, and long communication distances^4^,^5^. Furthermore, the center operating frequency and bandwidth are key factors in the design of RF antennas with specific band requirements, such as those used in industrial, scientific, and medical (ISM) devices, wireless medical telemetry services (WMTS), and medical implant communications services (MICS). In general, the frequency response of an antenna is easily controlled by changing its structural dimensions, allowing multifunctional devices containing RF antennas that accept wide frequency ranges and can act as strain sensors. This concept has been applied in smart skins^6^,^7^.

Among the various antenna electrode materials, graphene, a two-dimensional conducting material consisting of carbon atoms arranged in a hexagonal lattice, has attracted a great deal of interest due to its high electron mobility, unique mechanical flexibility and ease of implementation^8^,^9^,^10^,^11^. In particular, the ability of graphene to support surface plasmon polaritons (SPP) has facilitated the creation of micron-sized, graphene-based antennas for use at very high resonant frequencies^12^,^13^,^14^. Graphene made via chemical vapor deposition (CVD) is widely used as a base material for wireless communications components. Although passive CVD graphene antennas boast low power consumption due to their high conductivity and nanometer dimensions, their operating frequency range is limited by difficulties in obtaining samples up to several centimeters^15^,^16^,^17^. In these regards, multilayer graphene derived from graphite is a good candidate for fabricating resonant structures at frequencies ranging from the microwave to the terahertz band. These multilayer films can be made using convenient, low-cost, and scalable solution-processing techniques^18^,^19^. Moreover, the surface wave propagation that affects radiation frequency can be controlled by tailoring the size and shape of the antenna by implementing simple patterning methods during fabrication. This facilitates both integration and the attainment of low actuation voltages^20^,^21^,^22^. However, antennas made in this fashion had low efficiencies, relative to metallic analogs, at the lower frequencies required in industrial applications. This shortcoming was ascribed to the lower electrical conductivity of the carbon-based materials^23^. Therefore, the development of an efficient and facile strategy for fabricating highly omnidirectional, frequency-controllable carbon-based antennas with high radiation efficiencies is still in demand.

Herein, we describe a novel route toward the fabrication of multifunctional carbon/polyaniline (C/PANI)-based two dimensional (2D) and three dimensional (3D) monopole antennas via screen printing and a one-step, dimensionally confined hydrothermal process, respectively. To our knowledge, this is the first experimental study to demonstrate the fabrication of highly omnidirectional and frequency-controllable carbon-based antennas. Importantly, solvated C/PANI was fabricated via low-temperature interfacial polymerization. The high hydrophobicity, unique mechanical and electrical properties, and high crystallinity of the resulting polymer allowed the creation of highly efficient, high-frequency 2D and 3D monopole antennas with omnidirectional electromagnetic responses. Stress-strain and electromechanical stability tests were performed to compare the mechanical properties of the C/PANI thin film with those of pristine carbon and PANI films. The relationships between electrical conductivity, permittivity, and crystallinity were also investigated. In addition, both C/PANI films and fibers were evaluated as monopole antennas in terms of mean resonance frequency, radiation efficiency, and omnidirectional response pattern.

## Results

### Fabrication of carbon, PANI and C/PANI-based 2D monopole antennas

**Figure 1:**
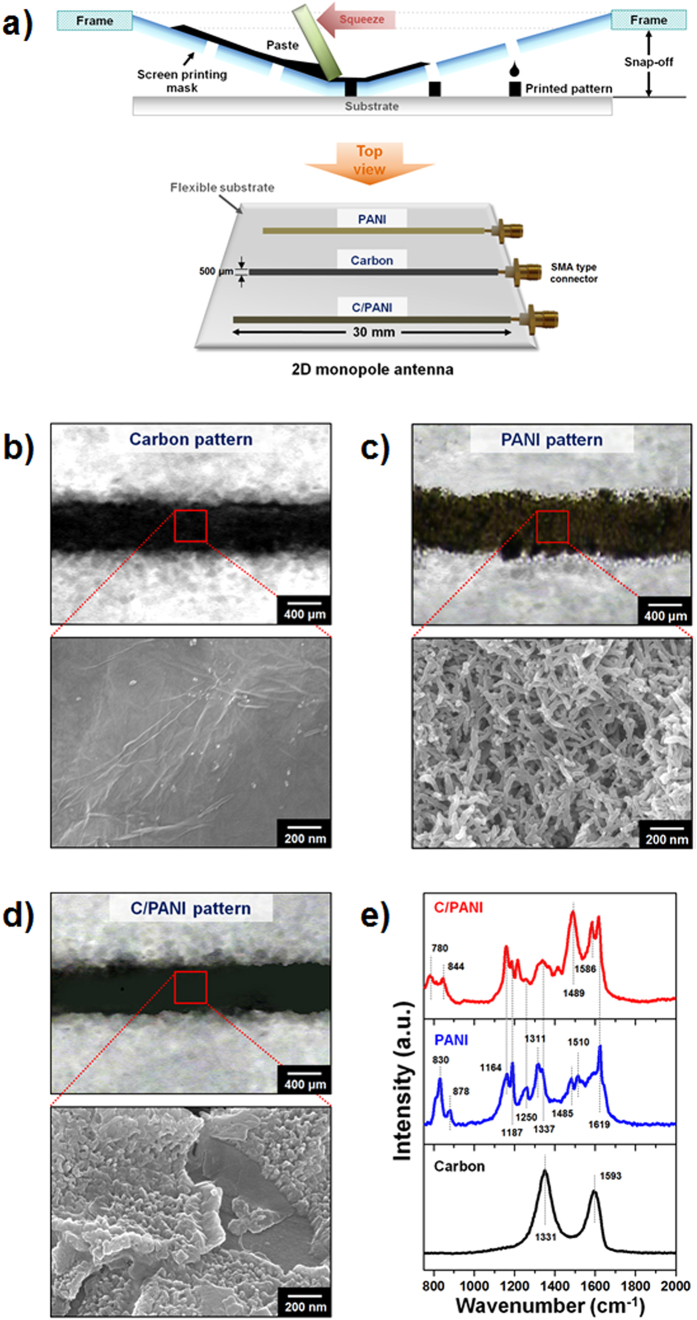
(**a**) Fabrication of conductive films on flexible substrates via screenprinting and a top-view schematic of various 2D monopole antennas with an SMA connector. Optical and FE−SEM micrographs show the surface morphology of (**b**) carbon, (**c**) PANI, and (**d**) C/PANI-based straight lines (*ca.* 500 μm × *ca.* 30 mm). (**e**) Raman spectra of carbon, PANI, and C/PANI thin films. Raman samples were prepared by screen-printing three conductive inks onto a Si wafer.

Solvated C/PANI in m-cresol/chloroform has been synthesized via low-temperature interfacial polymerization with dedoping and redoping processes^24^,^25^. In this experiment, this strategy was employed, using the polymer solution as a conductive ink in screen printing. This allowed the fabrication of 2D antennas. The procedure used to create C/PANI line patterns on a flexible substrate by screenprinting and a schematic structure of a 2D C/PANI-based monopole antenna are shown in Fig. 1a. The attached mask on photo paper formed an open mesh area that allowed the conductive ink to form a sharp-edged image as it transferred to the substrate. In this way, straight-line patterns (30 mm × 500 μm) were easily fabricated on photo paper. PANI thin films have been obtained by interfacial polymerization without carbon. After, a carbon thin film can be fabricated using graphene oxide (GO) ink that is then reduced by hydrazine vapor^26^,^27^,^28^,^29^. The optical and FE−SEM micrographs in Fig. 1b,c, respectively, show that carbon thin film exhibited a wrinkled and silk-like two-dimensional morphology that was on the micrometer scale. PANI was uniformly polymerized on the surface of the graphene sheets, and the average thickness of the hybrid thin film was assumed to be *ca.* 2.16 μm (Fig. S1). Furthermore, the pattern obtained with carbon and C/PANI is clearer than that obtained with PANI. This may result from the effects of graphene grains on the polymer domain structure (Fig. 1d). In other words, stacks of large-domain, planar graphene structures resulted in high-resolution patterning in the overlaying C/PANI thin film.

Raman spectra of the C/PANI films contain peaks that are characteristic of graphene and the emeraldine salt (ES) form of PANI (Fig. 1e)^30^,^31^,^32^,^33^. The increased intensity of quinoid ring-related vibrations and the C = C stretching mode of the quinoid ring at 1586 cm^−1^ indicated strong inter-molecular π–π stacking interactions between the basal planes of graphene and the quinoid rings of the PANI backbone^34^,^35^. The overlapping of P_z_ orbitals between the quinoid rings of PANI and aromatic surface of graphene would result in strong vibrational peaks. Detailed peak assignments are presented in Table S1.

### Mechanical, electrical and dielectric properties of fabricated 2D monopole antennas

**Figure 2:**
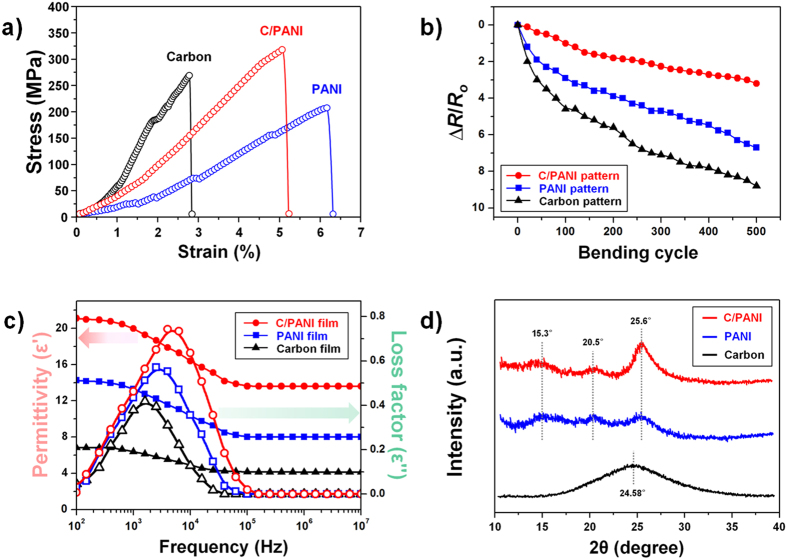
(**a**) Typical stress-strain curves under tensile loading, (**b**) electrical-resistance changes upon repeated bending, (**c**) dielectric constants and (**d**) XRD diffractograms are shown for carbon, PANI, and C/PANI thin films. Mechanical measurements were performed at a strain rate of 1 mm s^−1^. Loss factors were obtained by differentiation of the permittivity values. Powdered samples were used in XRD analyses.

Representative stress−strain curves of the synthesized thin films, obtained using UTM, are shown in Fig. 2a. The carbon and PANI thin films had tensile strengths of 264 and 208 MPa and moduli of 7.13 and 4.33 GPa, respectively. Significant improvements in tensile strength and modulus were observed with the C/PANI thin film, which exhibited a tensile strength of 315 MPa and a modulus of 10.05 GPa (Table 1). Compared to carbon and PANI thin films, the elongation at break was higher for the C/PANI thin film (*ca.* 6.31%). The work of extension to toughness was assumed to be *ca.* 8.74 MJ m^−3^ for the C/PANI thin film, which is slightly higher than that of pristine PANI thin films (*ca.* 6.93 MJ m^-3^). These results suggest that strong interactions between the graphene sheets and PANI enhanced the mechanical strength of the C/PANI thin films.

The electromechanical stability of the samples was assessed in electrical fatigue tests using an external force. The resulting sheet resistance change is expressed as *ΔR*/*R*_*o*_ = (*R*−*R*_*o*_)/*R*_*o*_, where *R* and *R*_*o*_ are the measured and initial sheet resistances, respectively. The *R*_*o*_ values of carbon, PANI, and C/PANI thin films were *ca.* 35, 2.1, and 0.6 Ω sq^−1^, respectively. Figure 2b shows the change in surface resistance as a function of bending cycle. When the samples were released from bending after 500 cycles, the *ΔR*/*R*_*o*_ values of C/PANI, PANI, and carbon thin films increased by about 3.2, 6.7, and 8.8, respectively. This indicates a high degree of structural stability in the C/PANI thin film. In contrast, the pristine carbon thin film cracked and fissured under an external force. The above results indicate that line-patterned thin films may be applicable as 2D monopole antenna electrodes.

Prior to implementation as antennas, the dielectric constants of the various samples were determined. The dielectric constant of a material affects the speed of propagation of an electromagnetic wave through that material. Electrical permittivities (*ε′*) and loss factors (*ε*″) were determined as shown in Fig. 2c. The permittivity at lower frequencies was always greater than that at higher frequencies and relaxation slopes (*α*_*a*_) mainly overlap at 1 kHz. Thus, the C/PANI thin films had significantly enhanced permittivities of 13.4 compared to those of carbon (4.0) and PANI (7.8) thin films. In addition, the dielectric strengths (*Δε*) of the C/PANI thin films were greater and relaxation times (*τ*_*m*_) were shorter (Table 1). It implies that highly reversible conformational rearrangements of molecules in the interior of the C/PANI films increased the stored energy in opposition to the external electric field^36^,^37^. In electromagnetics, permittivity is strongly influenced by the geometry of the materials through interfacial interactions between mediums. For this reason, XRD analyses were conducted to investigate the crystalline structures of the thin films (Fig. 2d). The diffractogram of pristine carbon presented one broad peak at 24.58° (*d*-spacing = *ca.* 3.62 Å). In contrast, three peaks were observed in the diffractogram of PANI at 2*θ* = 15.3, 20.5 and 25.6° (*d*-spacing = *ca.* 5.8, 4.3 and 3.5 Å) on a broad background, indicating a semi-crystalline structure. The diffractogram of C/PANI was dominated by a peak at 25.6° and the broad background was minimal. This indicates a relatively crystalline phase originating from planar chain conformations and strengthened stacking along a specific direction. This phenomenon induced extensive three-dimensional delocalization of charge, leading to the observed enhancements in electrical conductivity and permittivity^38^,^39^.

### Return loss and radiation properties of fabricated 2D monopole antennas

**Figure 3:**
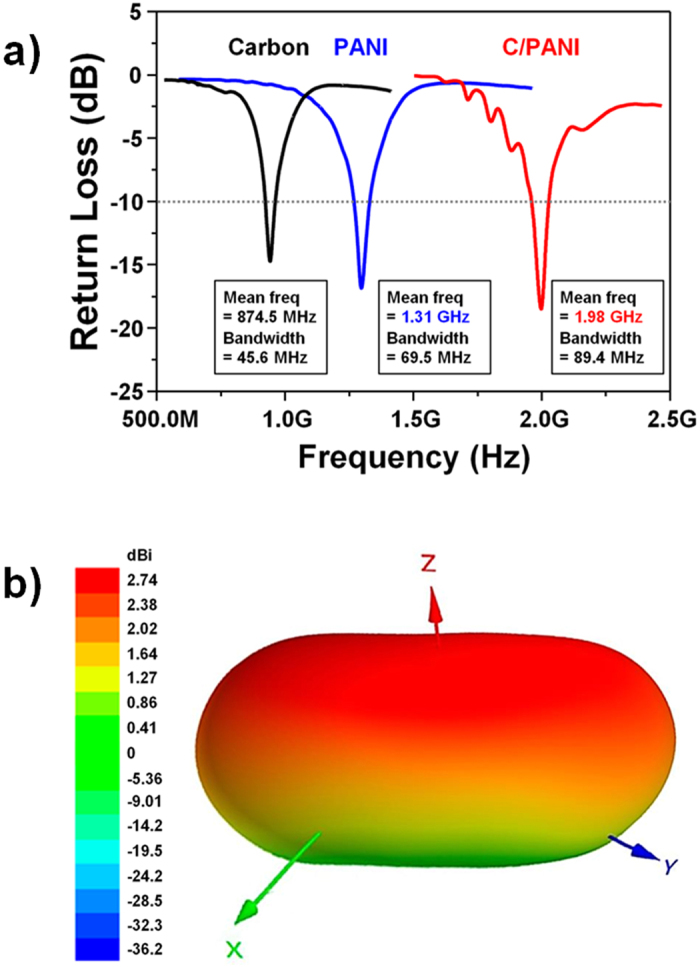
(**a**) The measured return loss curves are given for 2D monopole antennas composed of carbon, PANI, and C/PANI-based electrodes. (**b**) The simulated radiation pattern of a C/PANI-based 2D monopole antenna is shown using Ansoft HFSS.

Based on the above analyses, three different line patterns were used to make the electrodes for 2D monopole antennas. Generally, the performance of an antenna is evaluated using its mean frequency and voltage standing wave ratio (VSWR) or return loss (RL), which is indicative of transmitted power efficiency^40^,^41^. It refers to the loss of power due to impedance mismatching, which is designing the input impedance of an electrical load or the output impedance of its corresponding signal source to maximize the power transfer or minimize signal reflection from the load. Carbon, PANI, and C/PANI-based 2D monopole antennas had mean frequencies of 874.5 MHz, and 1.31 and 1.98 GHz, respectively (Fig. 3a). The VSWR (1.27) and RL (18.5) values of the C/PANI-based 2D monopole antennas indicated an increase in transmitted power efficiency of 98.6%, and the simulated values were also shown in Table 2. The large differences in mean frequency can be ascribed to differences in the permittivities of the antenna materials. In general, the speed of an electromagnetic wave propagating through a given medium is given as
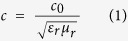
where *c*_*0,*_ *ε*_*r*_, and *μ*_*r*_ are the speed of light in free space, and the relative permittivity and permeability of the medium, respectively. Therefore, an increase in permittivity results in a decrease in the propagation speed of an electromagnetic wave in that medium. This then results in a decreased wavelength according to
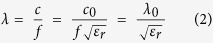
where *f* and *λ*_*0*_ are the frequency and wavelength of a plane wave in free space, respectively. The power gain, defined as the ratio of the power produced by the antenna from a far-field source on the antenna beam axis to the power produced by a hypothetical lossless isotropic antenna, of three types of 2D monopole antennas was simulated. Power gain, a metric of performance, combines the antenna directivity and radiation efficiency. Especially, radiation efficiency indicates of the ohmic/dielectric power loss in the antenna itself, and thus it is a measure of the radiating electromagnetic energy that propagates into the surrounding space through the antenna. Figure 3b shows that the C/PANI thin films acted as omnidirectional monopole antennas with relatively low gain along the substrate direction with a resultant interruption in wave propagation. The simulated radiation properties of 2D monopole antennas made from the various materials used in this study are summarized in Table 3. Compared to the carbon thin film antennas, the C/PANI-based 2D monopole antenna exhibited a peak gain of 1.49 dBi with a higher radiation efficiency of 87.83%. This is similar to the gain of a dipole antenna. These data suggest that the stronger mechanical strength, and higher permittivity and conductivity of the C/PANI thin films resulted in 2D monopole antennas with greater efficiency, higher resonance frequencies, and omnidirectionality.

### Synthesis of C/PANI-based 3D monopole antenna

**Figure 4:**
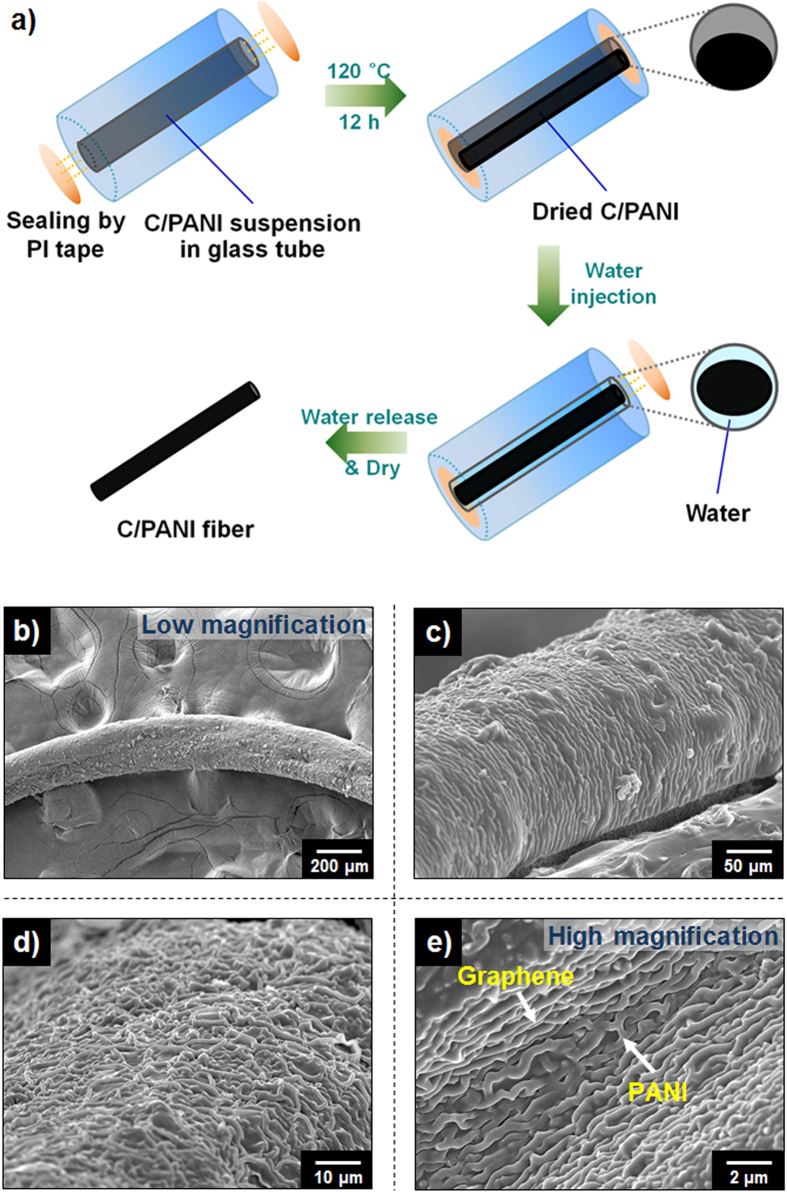
(**a**) A schematic illustration of the method used to synthesize C/PANI fibers in a glass reaction pipe with an inner diameter of 500 μm. The two ends of glass tube were sealed with PI tape prior to heating. Free-standing C/PANI fibers were obtained by releasing them from the reaction pipe by an injection of water and air drying. (**b–e**) Representative FE−SEM micrographs show the synthesized free-standing C/PANI fibers (width *ca*. 250–300 μm) at low and high magnification.

**Figure 5:**
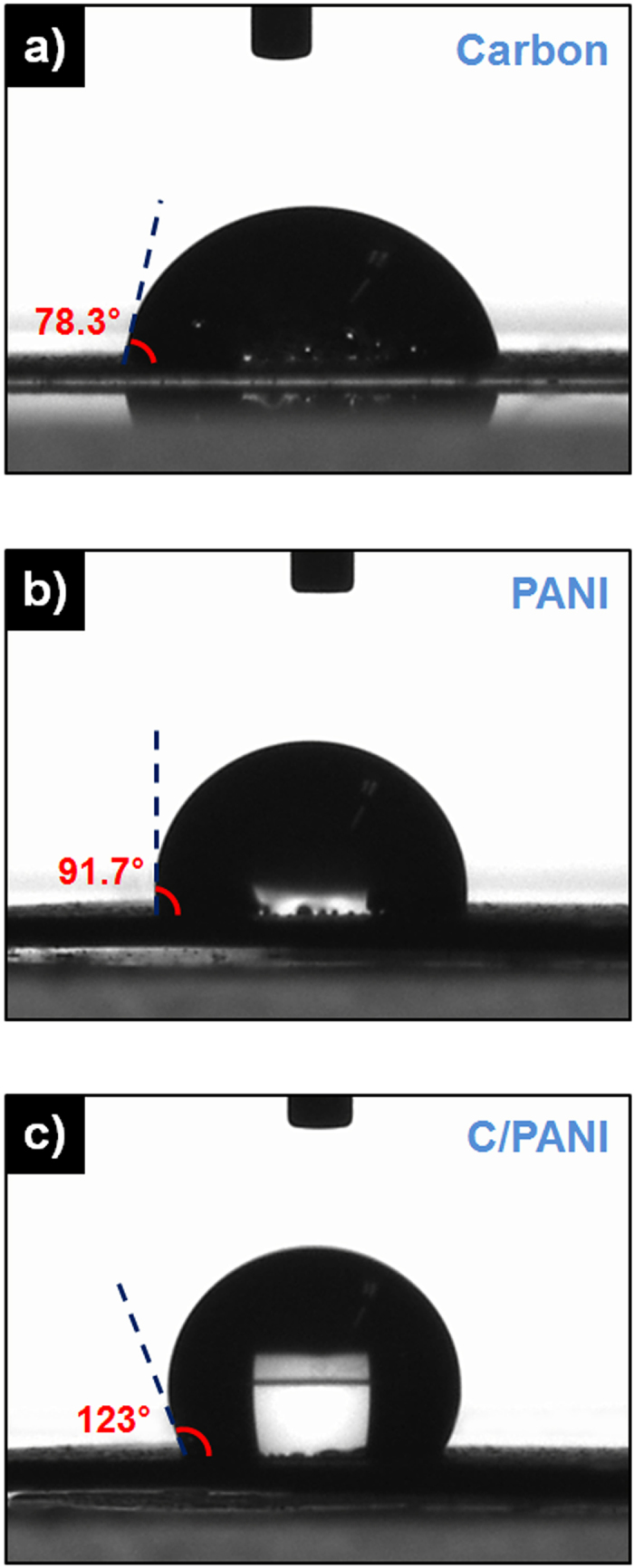
Water contact angles on the surface of (a) carbon, (b) PANI, and (c) C/PANI thin films. All samples were transferred onto flexible photo paper.

In addition, a facile one-step, dimensionally confined hydrothermal strategy was employed to fabricate C/PANI fibers (Fig. 4a). A glass pipe with an inner diameter of 0.5 mm was used as the reactor. Solvated C/PANI solution was injected into the glass pipe, which was then sealed at both ends with polyimide (PI) tape and heated to 120 °C for 12 h. Water was then injected into the pipe to delaminate the C/PANI. This process, which depended on the high hydrophobicity of the C/PANI (Fig. 5), released a C/PANI fiber matching the inner pipe geometry. This process was not as applicable to the synthesis of carbon or PANI fibers due to the lower hydrophobicity and lower mechanical strength of those materials. Figure 4b–e show the synthesized free-standing C/PANI fibers at low and high magnification. The fibers have diameters of *ca.* 250–300 μm. The approximate 50% decrease in diameter relative to the newly released fibers is due to solvent loss and associated shrinkage. This drastic contraction in diameter during the drying process produces strong surface tension forces that result in spontaneous orientation and dense packing of C/PANI along the main axis of the fibers. Fiber diameter and length can be controlled by using a reaction pipe with a predesigned length and inner diameter.

### Return loss and radiation properties of synthesized C/PANI-based 3D monopole antenna with different length

**Figure 6:**
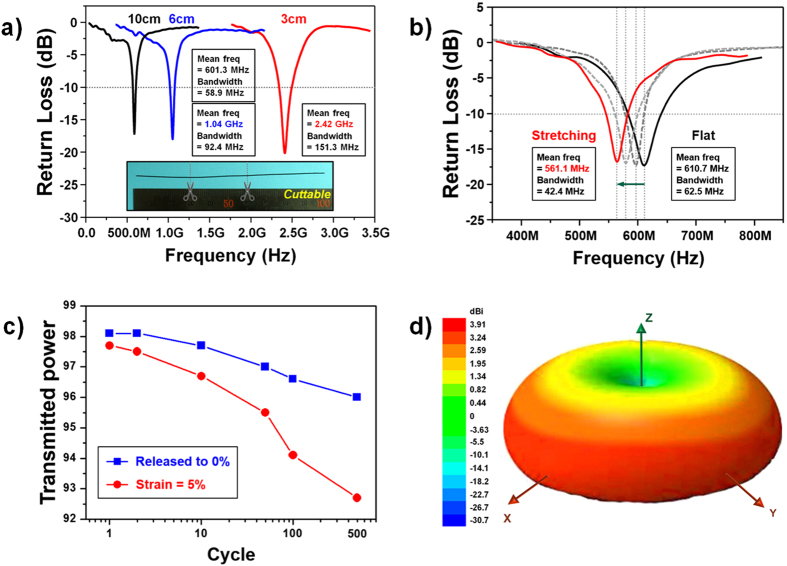
The measured return loss curve of a 3D monopole antenna composed of a free-standing C/PANI fiber is shown (a) as a function of fiber length and (b) after stretching. The inset in (**a**) shows a free-standing, 10-cm C/PANI fiber-based monopole antenna. The length of these C/PANI fiber-based monopole antennas could be controlled by cutting them with scissors. The 10-cm fibers were stretched by 1, 3, and 5% along the *x*-axis. (**c**) Changes in transmitted power are shown as a function of stretching cycle and (**d**) the simulated radiation pattern of C/PANI-based 3D monopole antennas is shown.

C/PANI fibers 10 cm in length were used as electrodes in 3D monopole antennas. Note that the length of these antennas can be adjusted by simply cutting the fibers with scissors. C/PANI-based 3D monopole antennas 3, 6, and 10 cm in length had center frequencies of 2.42, 1.04 GHz, and 601.3 MHZ, respectively (Fig. 6a). In general, resonant frequency decreased with increased antenna length. The high transmitted power efficiency (99.1%) of the 3-cm C/PANI-based 3D monopole antennas results from the high conductivity of the C/PANI, and the simulated RL value were also shown in Table 4. Furthermore, resonant frequencies decreased from 610.7 to 561.1 MHz as the 3D antennas were stretched to 1, 3, and 5% *x*-axis elongation from their original length of 10 cm (Fig. 6b). In addition, transmitted power efficiency decreased to 92.7% after 500 strain cycles. This demonstrates a high degree of structural stability in the C/PANI fibers when subject to an external force (Fig. 6c). The synthesized C/PANI-based 3D monopole antennas exhibited changes of less than 2% in their various properties over 3 months of storage in ambient conditions. Omnidirectional C/PANI-based 3D monopole antennas with high peak gains (3.60 dBi), directivity (3.91 dBi), and radiation efficiency (92.12%) were simulated (Table 5). These enhancements in these factors relative to those of 2D monopole antennas can be attributed to the high packing density and aspect ratio of the C/PANI fibers and the removal of the flexible substrate (Fig. 6d). However, stretching the C/PANI fibers resulted in a deterioration of performance due to a lowering in their mechanical strength, electrical conductivity and permittivity.

In general, high transmitted power is not only related to high conductivity but also the dimensions. In our experiment, the electrical conductivity of C/PANI-based thin film and fiber with 30 mm length was similarly *ca.* 906 S/cm. For this reason, the radiation efficiency of the C/PANI-based 3D monopole antenna without substrate was measured compared to that of the 2D monopole antenna to analyze the reduction rate in the efficiency by substrate loss (Table 6). As a result, the measured antenna properties such as peak directivity, gain and radiation efficiency were comparable to the simulated antenna properties. Most of all, the 3D antenna, compared with the 2D antenna, has increased the peak directivity and gain by 1.12 dBi with higher radiation efficiency (83.17%), which indicated of the 6.17% reduction in the efficiency originated from the substrate loss. These results suggest that frequency-controllable and highly omnidirectional 3D monopole antennas can be easily fabricated using a one-step, dimensionally confined hydrothermal strategy and cutting the fibers to the desired length with scissors.

## Discussion

Based on above results and understandings, we demonstrate that screen printing and a one-step, dimensionally confined hydrothermal process are considered as an efficient and a facile strategy for fabricating highly miniature, size and dimension-controllable C/PANI-based antennas. We believe that strong π–π interactions between the basal planes of graphene and the quinoid rings of the PANI backbone and extensive three-dimensional delocalization of charge in crystalline phase cause the enhanced mechanical strength, electrical conductivity and permittivity of C/PANI-based electrode (Fig. 2). The unique properties allow the creation of highly efficient and high-frequency C/PANI-based monopole antennas. The center frequency and transmitted power efficiency of 2D monopole antenna are independently related to the permittivity and conductivity of antenna materials. The mean frequency of C/PANI fiber-based 3D monopole antenna is adjusted by simply cutting and/or stretching the fibers. This antenna attained high peak gain (3.14 dBi), high directivity (3.39 dBi) and radiation efficiency (83.17%), which are attributed the high packing density and aspect ratios of C/PANI fibers and the removal of the flexible substrate (Table 6).

Rapid advances in wireless communication using a RF antenna to transfer the information have recently led to the development of ubiquitous systems integrated into a single wireless platform to allow maximum connectivity. Extensive efforts such as multiband antenna with filters to improve the out-of-band noise rejection and frequency-reconfigurable antenna using complicated switches are currently underway to use multiple wireless modules integrated on the same device. Therefore, it is imperative to reduce the number of the required filters and switches. From the point of view, our frequency-controllable carbon-based antenna by adjusting the permittivity and structural dimension using screen printing and a one-step, dimensionally confined hydrothermal process is considered as a potential breakthrough in terms of multiradio wireless platforms because of its remarkable characteristics including low cost materials, simple device component and multiband configuration.

## Conclusions

In conclusion, a simple and effective strategy to fabricate frequency-controllable and highly omnidirectional C/PANI-based 2D and 3D monopole antennas was demonstrated via screen printing and a one-step, dimensionally confined hydrothermal process, respectively. The ability to control resonant frequencies and directivity by adjusting the permittivity and structural dimensions of these carbon-based materials will be useful in the development of carbon-based wireless networking communications devices for use on ISM bands.

## Methods

### Materials

The graphite flakes used in the majority of experiments were purchased from Sigma-Aldrich (Product Number 332461). Aniline, camphorsulfonic acid (CSA), and ammonium persulfate (APS) were purchased from Sigma-Aldrich. Hydrochloric acid, ammonia solution, and chloroform were purchased from the Samchun Chemical Co. The used substrate material was photo paper with *ca.* 50 μm thickness, and it has a relative permittivity (*ε′*) ranging from 2.5 to 3.0 and loss tangent (tan δ) ranging from 0.01 to 0.05 over an operating frequency range of 500 MHz to 2.5 GHz.

### Synthesis of GO and carbon powder

Unreduced GO was synthesized from natural graphite by a modified Hummers and Offeman method as originally presented by Kovtyukhova and colleagues. Synthesized purified GO suspensions were dispersed in water. Exfoliation of GO was achieved by sonication of the dispersion for 3 h to avoid nozzle blockage. The obtained brown dispersion was then washed with five cycles of centrifugation at 3,000 r.p.m. to remove any unexfoliated graphite oxide. The GO solution was dried in a vacuum oven to obtain a GO powder. The GO powder was dissolved in deionized water (5 mg/mL) and hydrazine monohydrate was added to the solution (the volume ratio of hydrazine monohydrate:water = 1:1000). This solution was heated at 90 °C for 1 h. After reduction, the graphene solution was passed through filter paper with an excess of deionized water and dried in a vacuum oven. The resulting material was stored in powder form until further use.

### Preparation of C/PANI and PANI thin films via screenprinting

The as-prepared carbon powder was added to an HCl solution (2.5 M, 40 mL) and sonicated for 24 h. Aniline monomer was then added with vigorous stirring. Chloroform (60 mL) was then added to the mixture to induce a phase separation (chloroform phase at the bottom, aqueous phase on the top). The initiator, APS (weight ratio APS:aniline = 1.2:1) in HCl solution (3.75 M, 16 mL), was added to the above bi-phase solution and stirred for 24 h at −40 °C, resulting in a low-temperature interfacial polymerization. After polymerization, the solution was centrifuged and dried. The feeding weight ratio of aniline to carbon was 10:1. Primary doped C/PANI powder was dispersed in an ammonia solution (1.2 M, 215 mL) and vigorously stirred for 24 h to de-dope the PANI chains on the graphene. The De-C/PANI powder was then mixed with the CSA (mole ratio of PANI:CSA = 2:1) to re-dope the PANI chains on graphene. The Re-C/PANI powder was added to the m-cresol/chloroform solution (volume ratio of m-cresol: chloroform = 7:3), stirred for 3 h, and then sonicated for 24 h. Solvated C/PANI could be used as a conductive ink in screen printing and C/PANI-based thin films (3 cm × 500 μm) were screen-printed onto photo paper. Solvated PANI was also synthesized via an identical low-temperature interfacial polymerization with subsequent dedoping and redoping. PANI thin films were also fabricated using screenprinting.

### Patterning of carbon-based thin films by screenprinting and hydrazine vapor deposition (VDP)

The exfoliated GO solution was screen-printed onto flexible photo paper according to predetermined designs. Subsequently, the printed films were cut to an appropriate size and placed in a VDP chamber containing hydrazine and ammonia solution. The carbon patterns were formed immediately at low temperature (90 ^°^C, 1 h).

### Fabrication of C/PANI fiber

A glass pipe with an inner diameter of *ca.* 0.5 mm was used as the reaction vessel. The pipe was filled with solvated C/PANI and the ends were sealed with polyimide (PI) tape prior to heating at 120 ^°^C for 12 h. The dried C/PANI fibers were removed from the glass pipe by an injection of water. The resulting C/PANI fibers were *ca.* 250–300 μm in diameter.

### Characterization

Field-emission scanning electron microscope (FE-SEM) micrographs were acquired with a JSM-6700F microscope (JEOL, Tokyo, Japan) at an acceleration voltage of 10 keV. Optical micrographs were acquired using a Leica DM2500 P microscope. Raman spectra were obtained with a Jobin-Yvon T64000 spectrometer. Raman samples were screen-printed onto Si wafers (Sunmechanix, SM-S550). X-ray diffraction (XRD) analyses were performed on a MAC Science Co. M18XHF-SRA using powdered samples. Static contact angles were measured on a DSA 100-drop shape analysis system (Kruss GmbH). Permittivity was measured using a Solatron SI 1260 impedance/gain-phase analyzer with a Solatron 1296 dielectric interface. Measurements of electrical resistance were performed with a Keithley 2400 sourcemeter at 25 ^°^C using a four-point probe method. Sheet resistance was measured at 10 different locations on the samples and is given as an average value. RFID antenna characteristics were determined using an E5071B ENA RF network analyzer from Agilent Technologies from 300 kHz to 8.5 GHz. Impedance was plotted on a Smith chart by first normalizing to the characteristic impedance of the system (50 Ohms). In this experiment, C/PANI fiber for 3D monopole antenna could be directly connected to the SMA type antenna connector without external matching network using feed line. Furthermore, the rectangular ground plane using copper foil for all monopole antennas was designed to be 108.0 mm × 87.6 mm, and it was connected to the power supply ground terminal.

## Additional Information

**How to cite this article**: Shin, K.-Y. *et al.* Highly Omnidirectional and Frequency Controllable Carbon/Polyaniline-based 2D and 3D Monopole Antenna. *Sci. Rep.* **5**, 13615; doi: 10.1038/srep13615 (2015).

## Supplementary Material

Supplementary Information

## Figures and Tables

**Table 1:** Mechanical properties and dielectric parameters of carbon, PANI and C/PANI thin film.

Sample	Modulus^a^ (GPa)	Tensile strength^a^ (MPa)	Elongations at break^a^ (%)	Toughness^a^ (MJ m^−3^)	ε′^b^	Δε^b^	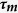 (μs)^c^
Carbon	7.13	264	2.89	3.45	4.0	2.8	100
PANI	4.33	208	6.31	6.93	7.8	4.6	63
C/PANI	10.05	315	5.20	8.74	13.4	7.6	25

^a^Values were obtained by stress−strain curve using UTM.

^b^Values were calculated by Havriliak-Negami and Fourier transforms relationship.

^c^Values were obtained by interfacial polarization response according to the relaxation time.

**Table 2:** VSWR, RL and transmitted power values of carbon, PANI and C/PANI thin film-based 2D monopole antennas.

Sample	Simulated results^a^			Measured results^b^		
	VSWR	RL (dB)	Transmitted Power (%)	VSWR	RL	Transmitted Power (%)
Carbon	1.40	15.8	97.2	1.44	14.9	96.7
PANI	1.28	18.2	98.5	1.34	16.8	97.9
C/PANI	1.23	19.7	98.9	1.27	18.5	98.6

^a^These values were obtained by Ansoft HFSS (S11).

^b^This value was acquired in the E5071C ENA RF Network Analyzer of Agilent Technologies (S11).

**Table 3:** Simulated peak directivity, gain and radiation efficiency values of carbon, PANI and C/PANI thin film-based 2D monopole antennas.

Sample	Peak directivity^a^ (dBi)	Peak gain^a^ (dBi)	Radiation efficiency^a^ (%)
Carbon	1.38	0.92	66.81
PANI	2.11	1.70	80.34
C/PANI	2.74	2.41	87.83

^a^These values were obtained by Ansoft HFSS.

**Table 4:** VSWR, RL and transmitted power values of C/PANI fiber-based 3D monopole antennas with different length.

Length (cm)	Simulated results^a^			Measured results^b^		
	VSWR	RL (dB)	Transmitted Power (%)	VSWR	RL	Transmitted Power (%)
3	1.14	23.7	99.6	1.22	20.1	99.1
6	1.16	22.6	99.5	1.29	17.9	98.4
10	1.18	21.7	99.3	1.32	17.2	98.1
10.5 (stretching)	1.20	21.0	99.2	1.36	16.3	97.7

^a^These values were obtained by Ansoft HFSS (S11).

^b^This value was acquired in the E5071C ENA RF Network Analyzer of Agilent Technologies (S11).

**Table 5:** Simulated peak directivity, gain and radiation efficiency values of C/PANI fiber-based 3D monopole antennas with different length.

Length (cm)	Peak directivity^a^ (dBi)	Peak gain^a^ (dBi)	Radiation efficiency^a^ (%)
3	3.91	3.60	92.12
6	3.87	3.49	90.35
10	3.64	3.34	91.65
10.5 (stretching)	2.28	1.58	69.44

^a^These values were obtained by Ansoft HFSS.

**Table 6:** Measured peak directivity, gain and radiation efficiency values of C/PANI-based 2D and 3D monopole antennas with a 30 mm length.

Sample	Peak directivity^a^ (dBi)	Peak gain^a^ (dBi)	Radiation efficiency^a^ (%)
2D antenna	2.25	2.02	77.00
3D antenna	3.39	3.14	83.17

^a^These values were measured in the anechoic chamber at the Electromagnetic Wave Technology Institute (Korea).
