# Supplementary material for: Highly Omnidirectional and Frequency Controllable Carbon/Polyaniline-based 2D and 3D Monopole Antenna
Source: Sci Rep. 2015 Sep 4;5:13615. doi: 10.1038/srep13615 (PMC4559896; doi:10.1038/srep13615)
Supplement: Supplementary Information [file srep13615-s1.pdf]

Supplementary Information for:

Highly omnidirectional and frequency controllable carbon/polyaniline-based 2D and 3D monopole antenna

Keun-Young Shin[1,2*], Minkyu Kim[1*], James S. Lee[1] and Jyongsik Jang[1,§]

[1]World Class University program of Chemical Convergence for Energy & Environment, School of Chemical and Biological Engineering, Seoul National University, 151-742, Korea

[2]A Photo-Electronic Hybrids Research Center, Korea Institute of Science and Technology (KIST), Seoul 136-791, Korea.

[§]Correspondence and requests for materials should be addressed to J.J. (jsjang@plaza.snu.ac.kr).

[*] E-mail: jsjang@plaza.snu.ac.kr
Tel.: +82-2-880-7069
Fax: +82-2-888-1604

1. SEM image for cross-section of C/PANI thin film

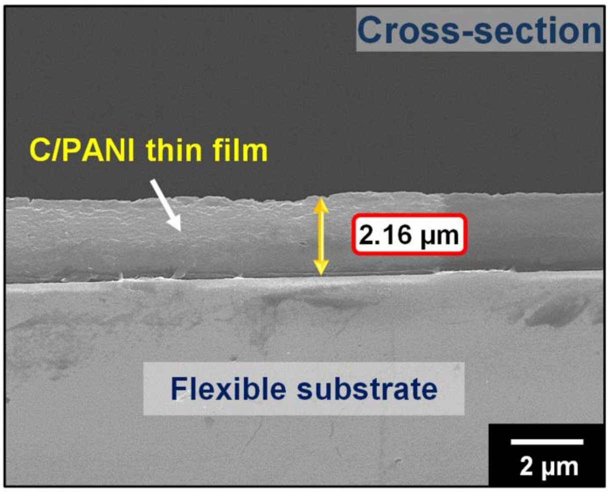

Figure S1. The SEM micrograph showing the cross-section of C/PANI thin film on the flexible substrate.

2. Raman peak assignments

The Raman spectra of carbon exhibit a high I_D/I_G ratio, suggesting that the reduction process increased the defect content or edge area of the graphene sheets. The relatively small dimensions of the graphene sheets may also account for the higher content of structural defects. The Raman spectra of PANI show distinct peaks corresponding to the C–C stretching of the para-distributed benzenoid ring, C=N stretching of the quinoid ring, C=N stretching of the quinoid ring, C–N•+ stretching in the polaron (cationic amine units of the benzenoid ring), C–N+ stretching in the bipolaron (cationic imine units of the quinoid ring), C–N stretching, C–H bending of the benzenoid ring, C–H bending of the quinoid ring, benzenoid ring deformation, and C–H bending of the quinoid ring and quinoid ring deformation at 1619, 1510, 1485, 1337, 1311, 1250, 1187, 1164, 878 and 830 cm^{-1}, respectively. After the aniline monomers were polymerized on graphene, the intensity of the quinoid ring-related vibrations increased, and the C=C stretching mode of the quinoid ring at 1586 cm^{-1} became apparent. This was indicative of strong inter-molecular π–π stacking between the basal planes of the graphene and the quinoid rings of the PANI backbone. The overlap of the P_z orbitals of the quinoid rings of PANI with the aromatic surface of graphene would result in strong quinoid ring peaks in the Raman spectrum of the Re-C/PANI film. Furthermore, a peak corresponding to C–N stretching in the emeraldine base (EB) form appeared at 1215 cm^{-1} along with two quinoid ring vibrations at 780 and 1489 cm^{-1}. The spectral changes indicate changes in the backbone structure of PANI to benzenoid rings and amine nitrogen atoms.

Table S1. Raman peak assignments of the C/PANI thin film.

Band positions[a] (cm^{-1})	Peak assignmets
1619	C–C stretching of the para-distributed benzenoid ring
1586	C=C stretching vibrational mode of the quinonoid ring
1510	C=N stretching of the quinonoid ring
1485	C=N stretching of the quinonoid ring
1337	C–N•+ stretching in polaron form (cationic amine units of the benzenoid ring)
1311	C–N+ stretching in bipolaron form (cationic imine units of the quinoid ring)
1250	C–N stretching
1215	C–N stretching in the emeraldine base (EB) form
1187	C–H bending of the benzenoid ring
1164	C–H bending of the quinonoid ring
878	Benzenoid ring deformation
830	C–H bending of the quinonoid ring
780	Quinonoid ring deformation

[a] For Raman analysis, the samples were deposited on Si wafer using screen printing

3. Dielectric constant measurement

Polarizability can be estimated by Havriliak-Negami and Fourier transfer relationship,

$$\varepsilon^* = \varepsilon' + i\varepsilon'' = \varepsilon_\infty + \frac{\Delta\varepsilon}{1+(i\omega\tau_m)^{1-\alpha}}$$

Where ε' is dielectric constant, ε'' is the dielectric loss factor, therefore, polarizability is as follows:

$$\Delta\varepsilon = \varepsilon_S - \varepsilon_\infty$$

Where ε_S is ε' as $\lim_{\omega\to 0}\varepsilon^*(\omega)$, and ε_∞ is ε' as $\lim_{\omega\to\infty}\varepsilon^*(\omega)$, calculated in Table 1.

Proper interfacial polarization response by relaxation time, is defined as follows:

$$\tau_m = \frac{1}{2\pi f_{max}}$$

Where f_{max} is the frequency of the loss peak, calculated in Table 1.
